# Mapping eGFP Oligomer Mobility in Living Cell Nuclei

**DOI:** 10.1371/journal.pone.0005041

**Published:** 2009-04-04

**Authors:** Nicolas Dross, Corentin Spriet, Monika Zwerger, Gabriele Müller, Waldemar Waldeck, Jörg Langowski

**Affiliations:** 1 Division Biophysics of Macromolecules, German Cancer Research Center (DKFZ), Heidelberg, Germany; 2 Biophotonique Cellulaire Fonctionelle, IRI, Parc de la Haute Borne, Villeneuve d'Ascq, France; 3 Division of Functional Cell Biology, German Cancer Research Center (DKFZ), Heidelberg, Germany; University of Birmingham, United Kingdom

## Abstract

Movement of particles in cell nuclei can be affected by viscosity, directed flows, active transport, or the presence of obstacles such as the chromatin network. Here we investigate whether the mobility of small fluorescent proteins is affected by the chromatin density. Diffusion of inert fluorescent proteins was studied in living cell nuclei using fluorescence correlation spectroscopy (FCS) with a two-color confocal scanning detection system. We first present experiments exposing FCS-specific artifacts encountered in live cell studies as well as strategies to prevent them, in particular those arising from the choice of the fluorophore used for calibration of the focal volume, as well as temperature and acquisition conditions used for fluorescence fluctuation measurements. After defining the best acquisition conditions, we show for various human cell lines that the mobility of GFP varies significantly within the cell nucleus, but does not correlate with chromatin density. The intranuclear diffusional mobility strongly depends on protein size: in a series of GFP-oligomers, used as free inert fluorescent tracers, the diffusion coefficient decreased from the monomer to the tetramer much more than expected for molecules free in aqueous solution. Still, the entire intranuclear chromatin network is freely accessible for small proteins up to the size of eGFP-tetramers, regardless of the chromatin density or cell line. Even the densest chromatin regions do not exclude free eGFP-monomers or multimers.

## Introduction

The accessibility to compartments and the global binding energy landscape faced by biomolecules are important parameters determining their function, and their quantification is an essential task for cell biology. While active transport in cells has its main role in the exchange between compartments, intracompartment mobility on the typical length scale of cells (some 10 µm) is mainly governed by Brownian motion [1]. Recent studies demonstrate that proteins show anomalous diffusion – i.e. a mean-square displacement whose time dependence is weaker than linear – in the cytoplasm [2] as well as in the nucleus of living cells [3]. This implies either geometrically obstructed or spatially confined motion [4]–[6]. The diffusion in the nuclei of living cells is affected by the distribution and the density of the intranuclear obstacles, the transient binding of the proteins to these obstacles, the local viscosity or active transport phenomena.

Chromatin is a binding target for many nuclear proteins implied in functions such as chromatin remodeling and repair [7], epigenetic regulation [8] or gene transcription [9]. Furthermore, since the chromatin chain fills 5 to 12% of the cell nucleus [10], it must be taken into account as a static obstacle even for non-binding molecules. Previous studies demonstrated the influence of the chromatin network on the diffusion of larger objects [11], [12], but its impact on the motion of smaller molecules has not been quantified so far. Since the diffusion of small proteins in the cell nucleus is central to their mechanism of action as well as to understanding nuclear architecture, we studied how diffusion of such macromolecules is affected by the chromatin network.

Diffusion in living cells can be quantified in several ways, most of them based on fluorescence measurements. Fluorescence correlation spectroscopy (FCS) is particularly suitable for characterizing mobility in the millisecond to second range. FCS measures fluorescence intensity fluctuations arising from the Brownian motion of fluorescent molecules into and out of a sub-femtoliter laser focus, or from transitions between fluorescent and non-fluorescent states. Nanomolar concentrations can be analyzed, compatible with protein studies at an endogenous expression level. This approach was first used in vitro by Koppel and coworkers [13]. From the early 2000s there has been increasing use of FCS for biological applications including living samples [4], [10], [14]–[18]. Since then some groups provided rigorous protocols for FCS measurements in order to avoid artifacts [19], [20] and adapted to the constraints of live samples [14], [21].

Here we will first present characterization and validation steps required for quantitative Fluorescence Fluctuation Microscopy (FFM) experiments [6]. FFM in our context is defined as the combination of FCS with confocal laser scanning microscopy (CLSM). This technique allows imaging of the spatial distribution of fluorescent molecules and probing their mobility at the locus of interest by precisely positioning the laser with the scanning unit.

The FFM procedure is particularly suited to quantify spatially varying protein mobility, e.g. as a function of chromatin density, using appropriate fluorescent reporters. For measuring chromatin density, we fused histone H2A with the monomeric Red Fluorescent Protein 1 (H2A-mRFP) and transfected it into various human epithelial cell-lines. Controls with DNA counterstaining showed that the distribution of such fluorescence-modified histones is equivalent to the distribution of the DNA in cell nuclei [22]–[25]. We chose mRFP over other red proteins for its advantages for *in vivo* measurements. It folds completely in cells at 37°C [26], is monomeric, does not aggregate and is not cytotoxic. For diffusion measurements, double live labeling is necessary. A good pair of autofluorescent proteins is given by combining eGFP [27] to the previously chosen mRFP. Since our goal was to quantify the diffusion of small molecules of various sizes, we decided to use eGFP mono-, di-, tri- and tetramers as mobility reporters.

Combining a rigorously characterized setup and an adequate reporter strategy, we could for the first time quantify the accessibility of the nuclear landscape and the diffusion coefficient of small molecules, up to eGFP tetramers, depending on the chromatin compaction level.

## Methods

### Cell lines

Adherent HEK293 (from human embryonal kidney), HeLa (from human cervix carcinoma), TP366 and T98G (both from human glioblastoma) cells were grown in a 5% CO_2_ humidified atmosphere at 37°C. They were passaged in phenol red free DMEM (Dulbecco's modified Eagles) and supplemented with 10% fetal calf serum (Invitrogen Life Technologies, Carlsbad, CA, USA) and 1% glutamine (Biochrom, Germany). For *in vivo* imaging and measurements, cells were cultured sub-confluent in Falcon flasks and then transferred to 32 mm cover slips in 6-well-plates. After 48 hours, the cover slips with the adherent cells were washed in Hanks' Balanced Salts (PAN-Biotech, Aidenbach, Germany) and the full culture medium was replaced by phenol red free DMEM containing only 2% of fetal calf serum. Then cells were double-transfected with the mammalian expression vectors using FuGene HD (Roche Diagnostics, Mannheim, Germany) in different proportions, and with different DNA concentrations, depending on cell-line and construct, following the manufacturer's recommendations. We chose to work with transiently double transfected cells instead of stable cell lines in order not to depend too much on the specific genotype of one transfected cell.

For FFM-measurements, the cover slips were mounted on a measurement chamber developed in our laboratory [28] allowing a working volume of 3 ml, 14 to 24 hours after transfection. The measurement chamber was placed on the stage of the FFM in an incubator compartment at 37°C, 5% CO_2_.

### Constructs

Restriction enzymes were purchased from MBI Fermentas (Vilnius, Lithuania).

PCR amplifications were performed using PCR Master Mix (Promega, Madison, WI, USA). PCR purification and gel extraction kits were purchased from Macherey-Nagel (Düren, Germany). Plasmids were cloned in *Escherichia coli XL10* (Stratagene, Amsterdam, Netherlands) and isolated using Maxi Prep kits (Macherey-Nagel, Düren, Germany), employed as proposed by the manufacturer.

The commercially available p-eGFP-N1 plasmids (Clontech, Saint-Germain-en-Laye, France) encode eGFP-monomers. The plasmids encoding the eGFP dimer, trimer and tetramer were a generous gift from Dr. M.M. Nalaskowski (Universitätsklinikum Hamburg-Eppendorf) and created following the method described in [29] and [30].

The human histone H2A gene was amplified by genomic PCR and inserted N-terminal of the enhanced cyan fluorescent protein (eCFP) into the promoter less plasmid peCFP-1 (Clontech). Upstream we inserted the HindIIIC fragment of simian virus 40 (SV40) in reverse direction, such that the fusion protein of 372 amino acid residues was expressed through the early SV40 promoter. In a second step, eCFP was replaced by mRFP1.

### Cell lysis, gel electrophoresis and Western-blot

Dishes containing a confluent layer of transfected HeLa cells after 14 to 24 hours were washed 3 to 4 times with 100 nM STE-Buffer and the cells collected. The concentrated cell suspension was treated with a Dounce homogenizer, the resulting lysates were cleared of the largest debris by centrifugation and further purified via Vivaspin PES centrifugal filters and concentrators (Sartorius Stedim Biotech, Aubagne, France) with appropriate membrane size.

The soluble protein extracts were treated with non-reducing 5× native gel loading dye without boiling and assayed on a 10% native polyacrylamide gel with 0.1% SDS and a 1× Tris-Glycine-running-buffer pH 8.8. The in-gel fluorescence from the non-reduced eGFP samples was detected using a Typhoon 9410 Variable Mode Imager (Amersham Biosciences, Piscataway, NJ) using the 488-nm laser line for excitation and collecting the fluorescence at a wavelength of 520 nm.

For the Western blot, the same soluble cell extracts as for the native fluorescence gels were boiled with 3× Laemmli sample buffer for 5 minutes. Protein samples (15 µl sample eGFP-monomer, 11.25 µl eGFP-dimer, 11.25 µl eGFP-trimer and 7.5 µl eGFP-tetramer) together with molecular weight marker (15 µl; Broad Range, New England Biolabs) were subjected to sodium dodecyl sulfate-polyacrylamide gel electrophoresis (SDS-PAGE) and electroblotted. The membrane was incubated with rabbit polyclonal Anti-GFP antibody (GTX26556, GeneTex, Inc.), diluted 1∶2000, and - as loading control - the same membrane was incubated with mouse monoclonal Anti-β-Actin antibody (A 5441, Sigma), diluted 1∶10000. As secondary antibodies peroxidase-conjugated AffiniPure Goat Anti-Rabbit and Goat Anti-Mouse, respectively, were used diluted 1∶5000 (Jackson ImmunoResearch). All immune reactions were carried out in 10 mM Tris–HCl, pH 8.0, 150 mM NaCl, 0.05% Tween-20 (TBST) with 5% dried milk at RT with washing steps in TBST.

### Fluorescence Fluctuation Microscopy (FFM)

We employed a laboratory-built setup, the Fluorescence Fluctuation Microscope (FFM) [31], which is a combination of Fluorescence Correlation Spectroscopy (FCS) and Confocal Laser Scanning Microscopy (CLSM). FFM combines an FCS module and a galvanometer mirror scanning unit attached to the side video port of an inverted IX-70 microscope (Olympus, Hamburg, Germany) with an UplanApo / IR 60× water immersion objective lens with a numerical aperture (NA) of 1.2 [31], [32].

Intracellular measurements were all performed at 37°C, in a 5% CO_2_ humidified atmosphere, in a incubator chamber (EMBL, Heidelberg, Germany) surrounding the whole microscope.

For fluorescence excitation, we used an argon-krypton laser from CVI Melles Griot (Bensheim, Germany), with the 488 nm line for eGFP and the 568 nm line for mRFP1. The emission from eGFP was detected from 515 to 545 nm and between 608 and 662 nm for mRFP1 with two avalanche photodiodes (APD) (SPCM-AQR-13, Perkin-Elmer, Wellesley, USA), after passing appropriate dichroic mirrors and filters for spectral separation and selection. FCS measurements were carried out at laser intensities from 5 to 9 kW·cm^−2^ for both laser lines. Laser-power was adjusted with the help of a polychromatic acousto optical modulator AOTF Nc (AA Opto Electronic, France), which allows precise control over the laser-power.

Using a home-made control software, we acquired confocal fluorescence images and randomly chose the FCS measurement points with either high or low chromatin density, but avoiding the nucleoli.

The signals coming from the APDs were fed into an ALV-5000/E correlator card (ALV Laser GmbH, Langen, Germany), where intensity fluctuations were recorded and their autocorrelation function simultaneously and almost in real-time, calculated.

Control temperature measurements were performed on a “The Cube & The Box” incubator (Life Imaging Services) using a USB TC-08 Thermocouple Data Logger (Pico Technology) or on a GP-168 incubator (EMBL, Heidelberg, Germany) coupled with a THERM 2290-2/3 multifunctional measuring instruments (Ahlborn Mess- und Regelungstechnik GmbH, Holzkirchen, Germany)

### Preparation of fluorophore solutions

Fluorophore solutions were diluted in de-ionized water and deposited into a homemade measurement chamber [28] before acquisitions using FFM microscope. Compared to disposable observation chambers, our chamber uses high quality coverslips permitting a better adjustment of the objective correction collar. The low variance in the coverslips width allows obtaining a similar optical path for both reference and biological samples. Two widely used fluorophores were tested: alexa488 and rhodamine B.

### In vitro measurements of eGFP-multimers

The same soluble protein extracts as for the native polyacrylamide gel were used for *in vitro* measurements of the eGFP-multimers. Measurements were carried out at 37°C, in a humidified 5% CO_2_ atmosphere. The same measurement chamber as for *in vivo* experiments was used, with a working volume of about 2 ml.

### Intranuclear data acquisition and sorting

For data acquisition, a confocal image of the mid-section of the whole cell was recorded using CLSM. On this image, up to 5 random positions were selected in the nucleus of the cell, avoiding the nucleoli. We then performed autocorrelation measurements of six runs of ten seconds each for every single measurement point. A second image was recorded after the completion of the FCS measurements in order to exclude measurements in which the cell moved during the experiment. The laser power was about 5 kW·cm^−2^ in order to minimize photobleaching as well as cell damages, stress and thus movements. The crosstalk from the red channel into the green and *vice-versa* was negligible. We studied at least 44 representative points for each construct in each cell line.

Before data analysis, a first selection was done on the basis of the recorded image: each cell that showed a motion between the first and the second picture was discarded. The second selection criterion was the study of the recorded data set for the histone fluorescence intensity channel: the recorded intensities should decrease regularly due to photobleaching effect. Data showing an abnormal behavior of the fluorescence intensity (e.g. a rapid decrease followed by a slow one, or even an increase of the signal intensity) were discarded as well, these being indicators for conformational changes in the cell nucleus. This procedure ensures that the recorded and analyzed fluorescence fluctuations are due to particle diffusion and not to cell movement or reorganization.

The setup was calibrated using Alexa 488 (Molecular Probes, Eugene, Oregon) for the green channel and Alexa 568 for the red one. The focal volume was determined for every work session using a 20 nM Alexa 488 solution, diluted in sterile de-ionized water.

For collecting the diffusions maps we used only one laser line (488 nm) to excite both eGFP and mRFP1. This was done in order to reduce the cell's exposure to laser energy and therefore allowed for a maximal number of measurement points in the cells. At 488 nm, mRFP1 still shows over 35% excitation efficiency compared to its maximum absorption at 584 nm [26], [33], which does not constitute a major drawback to the measurement other than a globally reduced intensity of the mRFP1 fluorescence.

### Data analysis

The red channel data gives the fluorescence intensity from the histones, and therefore the chromatin density. We normalized the data using the average value of the red channel for the whole nucleus, allowing comparison of cells that showed different overall expression of the tagged histone. For this normalization, we assumed that every cell from a given cell line contains the same amount of DNA and that it is equally distributed over different cross-sections in a cell. The autocorrelation curves of each individual measured point are then fitted by a normal diffusion model, as described in [34]. We chose to fit the data to a normal diffusion model rather to an obstructed diffusion model because it is more robust and allows the fit of two distinct diffusive fluorescent populations [4].

In the normal diffusion model, the mean square displacement <*x^2^*> depends linearly on the time *t*:
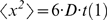
(1)where *D* is the diffusion coefficient.

To account for non-fluorescent processes and diffusing components of different sizes, we fitted the model function (Eq. 2) for two fluorescent diffusive components and one non-fluorescent component by the program *Quickfit*, written in our laboratory and based on the Marquardt-Levenberg algorithm [35].
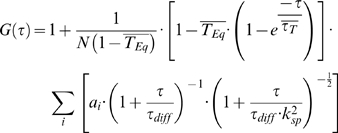
(2)


In equation (2), the nonfluorescent components are due to transitions of the fluorescent molecules into the triplet state, where 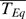 is the fraction of particles in this state, *a_i_* the relative fraction of each diffusive species, *N* the average number of particles in the focus volume and *k*_sp_ the structure factor, which depends on the focus volume and is given by:
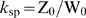
(3)where z_0_ is the axial and w_0_ the lateral dimension of the detection volume. The diffusion time ι_diff_ is related to the diffusion coefficient *D* by:
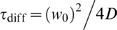
(4)


For all proteins in all cell lines, we obtained the fraction of the fast and the slow components as well as their respective diffusion times. From the latter we calculated diffusion constants using the measured diffusion times of Alexa 488 at 25°C and 37°C and its published diffusion constant [36]. The structure factor *k*_sp_ of the focus volume was determined using Alexa 488 in aqueous solution and was found to be consistent with the point spread function obtained with embedded fluorescent beads (200 nm diameter). In living cells, however, there is a refractive index mismatch that distorts the confocal volume and which cannot be corrected in a straightforward way. The effect of refractive index mismatch on FCS measurements was recently estimated by Müller et al. [37]. With the refractive index of the cellular environment of 1.36±0.004 [38], this mismatch would lead to an absolute error in diffusion coefficient of less than 10% at the focal depth used here (<20 micrometers). This is much smaller than the variations in diffusion time actually measured in our experiments, so it would be very unlikely that small variations in refractive index mismatch would cause the changes observed here. However, absolute values of the diffusion coefficients calculated from the measured diffusion times may be slightly underestimated. Since we compare diffusion coefficients which have all been measured inside living human cells under the same conditions, such deviations are not critical.

Diffusion maps were generated by interpolation from the FCS measurement points using the program *Origin* (OriginLab Corporation, Northampton, MA, USA).

### Diffusion simulations of eGFP-constructs

For computing the theoretical diffusion coefficients of the eGFP-constructs, assuming a rod shape, we used the latest available version of the public domain software *SEDNTERP* [39], [40]. *SEDNTERP* allows calculating the viscosity η and the density ρ for the used buffer as well as computing the partial specific volume, v̅ from the amino acid composition of the protein. It also allows building oligomers from monomers of a given composition, thus enabling to compute diffusion coefficients of rod-shape oligomers.

We first determined the diffusion coefficient of monomeric eGFP assuming a globular shape, using equation (5) and (6) with *V* as the volume of the protein, *M* the molecular weight, *v̅* the partial specific volume (from SEDNTERP), *N_a_* the Avogadro number and *r* the radius of the sphere, for calculating the radius of the sphere.
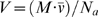
(5)

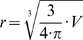
(6)


The diffusion coefficient of a spherical particle is given by the Stokes-Einstein equation:
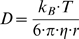
(7)where *D* is the diffusion coefficient, *k_B_* the Boltzmann constant, *T* the absolute temperature, *r* the radius of the sphere and *η* the viscosity of the solvent. The value for the radius, calculated from equation (6) was used in equation (7), with *η* equal to the viscosity of water, to get the theoretical diffusion coefficient of the globular shaped protein. The diffusion coefficient was then corrected for 37°C using
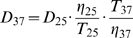
(8)and estimated for oligomers of molecular masses M_α_ and M_β_ (assumed spherical) with
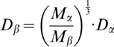
(9)


These correspond to the maximum possible diffusion coefficients for perfect spheres.

## Results

### Alexa 488 aqueous solution is optimal for focal volume calibration

**Figure 1:**
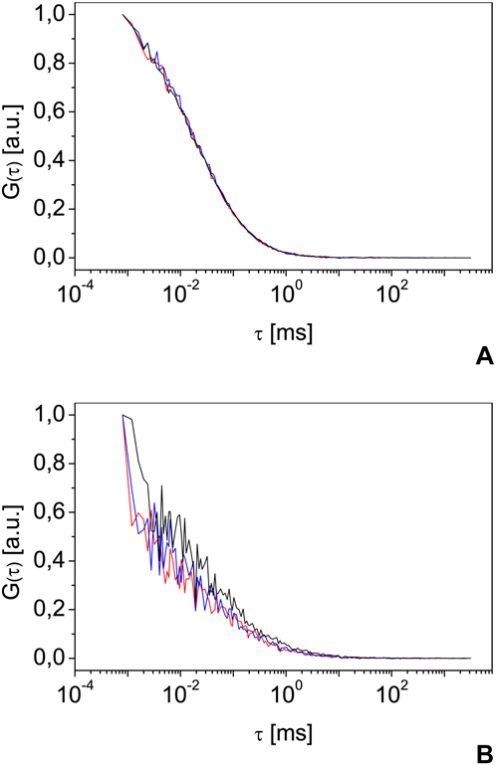
Alexa 488 and Rhodamine 6G as reference samples. Representative normalized autocorrelation curves measured on Alexa 488 (A) and Rhodamine 6G (B) 10 nM aqueous solution. The autocorrelation functions are normalized to 1 and expressed in arbitrary units (a.u.). All measurements were carried out under the same conditions, using a water immersion objective with a numerical aperture (NA) of 1.2.

Reliable FCS measurements depend on controlled and reproducible shape and dimensions of the focal volume. In one-photon excitation, the objective back-pupil should be slightly under-filled with a parallel laser beam [19]. The correction collar is then used to adjust for the cover slip thickness and thus to obtain a reproducible, artifact free Gaussian focal volume. The optimization of the excitation path as well as the determination of focal volume properties needs bright and photostable reference samples with a known diffusion coefficient. The fluorophores also should neither form aggregates nor stick to the cuvette walls. The buffer used varies from purified water tested for low fluorescence to more complex preparations [14]. In all cases, the chosen solution must be rigorously tested for its ability to be used for calibration. As an example of this necessity, figure 1 shows the differences between two commonly used calibration solutions. Alexa 488 diluted in water (fig. 1A) shows nearly perfectly overlapping autocorrelation curves, and thus highly reproducible results. Rhodamine 6G in water (fig. 1B) shows broadly varying autocorrelation curves, resulting from Rhodamine 6G molecules that aggregate or stick to the cuvette bottom and walls. These artifacts induce a reduced count rate and additional variable diffusion processes and differences between the calculated and measured number of molecules. Aqueous Rhodamine 6G solutions are therefore not recommended for FCS calibration, but aqueous Alexa 488 solutions allow reproducible focal volume estimation. From the known diffusion coefficient of 425 µm^2^ s^−1^ of Alexa 488 at 25°C [36], we can obtain from equation (4) a value for *w*_0_ and thus calculate the effective volume using the formula for a prolate ellipsoid:
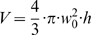
(10)where h is the long half-axis, obtained by multiplying the short half-axis *w*_0_ by the structure factor *k*_SP_ (see equation 3).

### Rigorous temperature control is needed for live cell experiments

Living human cells require a temperature of 37°C. Measurements carried out at room temperature could show artifacts due to the lower temperature and reduced metabolism of the cells. Therefore all diffusion measurements were performed at 37°C in an incubator to ensure proper diffusion analysis. Such systems need particular care for the calibration to be really efficient. In a liquid, diffusion of spherical particles is described by the Stokes-Einstein equation (Eq. 7). Water viscosity depends on the temperature according to an empirical law [41]:
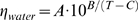
(11)with the parameters A = 2.414·10^−5^ Pa.s; B = 247.8 K; and C = 140 K.

**Figure 2:**
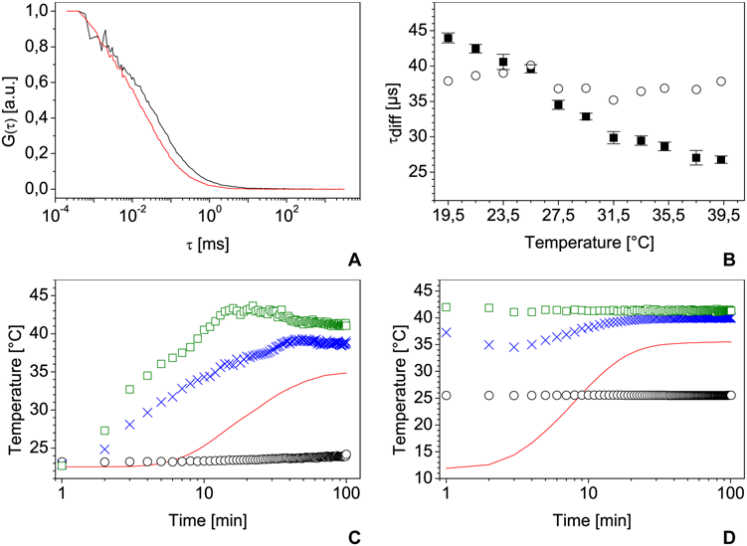
Highlight on temperature control. Normalized autocorrelation curves measured on Alexa 488 10 nM aqueous solution at 20°C (black curve) and 37°C (red curve) (A) and associated diffusion times for temperatures ranging from 19.5°C to 39.3°C with their standard deviation (B, squares) and diffusion times corrected for 25°C using temperature and water viscosity (B, circles). All measurements were done with the same laser power and a water immersion objective with a NA of 1.2. C and D: temperature measurements of room temperature (black circles), air temperature one centimeter behind the sample (blue crosses), air temperature at the incubator output (green squares) and temperature in the sample (red line). Temperature was measured from the incubator startup (C) or from the introduction of a 4°C sample introduction in a stabilized incubator (D).

Figure 2, A–B, shows this dependence on measurements performed on Alexa 488 at temperatures between 19.5 to 39.3°C. In this range, one can observe that the decrease of the diffusion time (Figure 2B, squares) can be approximate as linear as previously stated in [36]. Between each measurement step the system was allowed to heat up and stabilizes for at least one hour to ensure proper temperature and was adjusted for maximal count rate. This last step is very important due to the dilation of the mechanical parts (lenses, mirrors, FCS-module) with temperature. By correcting the diffusion times for 25°C for temperature and viscosity of water using equations (8) and (11) (figure 2B, circles) we obtain a mean value of ι_D,25_ = (37.5±1.4) µs which shows no major trend with temperature, in good agreement with previous measurements and theoretical values.

Since the setup must be calibrated before any FCS measurement, the reference sample used for the focal volume estimation must be measured under the same conditions as used for the live cell experiments. This is not trivial because most temperature controlled chambers or incubators measure the air temperature and not that of the solution in the sample holder, which is in contact with the microscope most of the time via an optical table. The temperature then stabilizes in the liquid later than elsewhere in the chamber: it takes typically 90 min for the sample but only 40 min for the incubator chamber to stabilize on the tested system (figure 2C). The sample temperature also often deviates from the nominal temperature selected in the control unit and needs some correction. Figure 2C presents such an example with a nominal temperature of 39°C in the incubator, leading to a liquid sample stabilized at only 35°C. Thus, a temperature calibration is needed, providing the stabilization time and correction to apply if necessary.

Another practical concern is that the reference sample and the live cells have antagonistic temperature requirements. The fluorophore is most stable at 4°C in the dark but it must reach 37°C before any reference measurement to calibrate live cells studies. In our case (figure 2D), this process takes around 60 minutes in an already stabilized incubator. Measurements performed after only a few minutes may underestimate the diffusion time by 50%.

### Focal volume distortion in FFM

**Figure 3:**
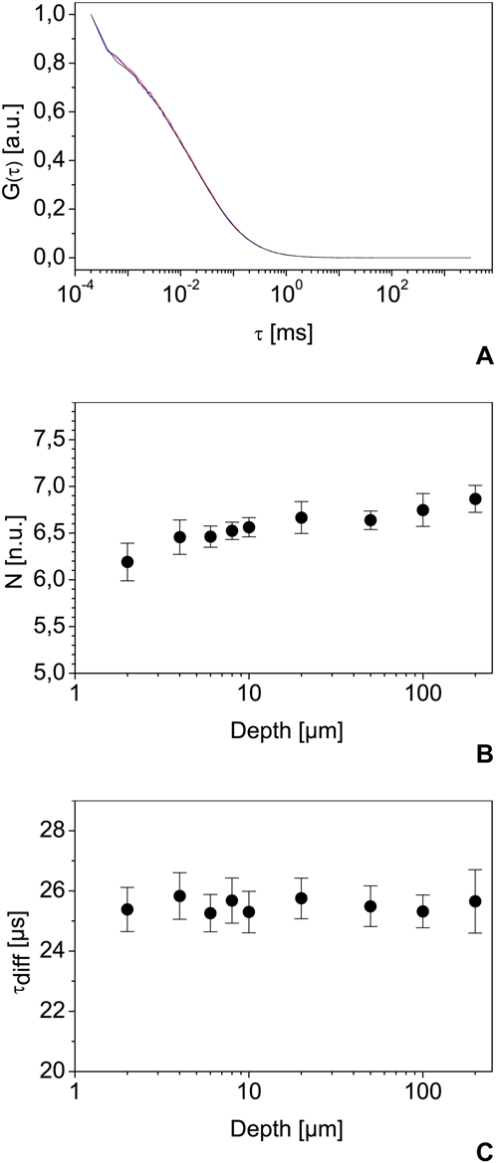
Effect of depth on FCS measurements. Representative normalized autocorrelation curves at 2 (red), 20 (blue) and 200 µm (black) depth (A) and average numbers of molecule in the focus N with their standard deviation at depths ranging from 2 to 200 µm (B) with their associated average diffusion times and standard deviation (C) measured on Alexa 488. The depth is measured relative to the top of the cover slip, using an inverse confocal system. Measurements were carried out using the same measurement chamber and laser power, using a water immersion objective with a NA of 1.2.

**Figure 4:**
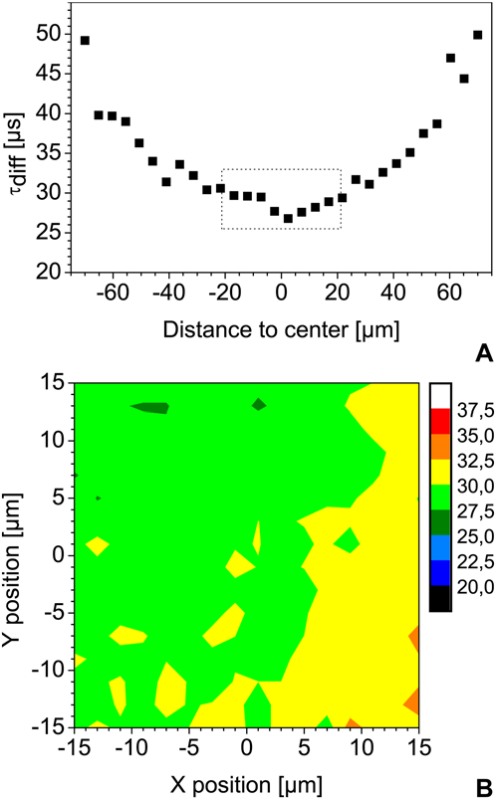
Effect of beam parking on reference measurements. (A) Diffusion times measured as a function of the diagonal distance from the center of the scanning field. An area of 100×100 µm gives a diagonal of approximately 140 µm. (B) Diffusion time map of the 30×30 µm central region. Each color step corresponds to a range of 2.5 µs in diffusion time ι. Both measurements were performed on Alexa 488 10 nM aqueous solution, using a water immersion objective with a NA of 1.2.

**Figure 5:**
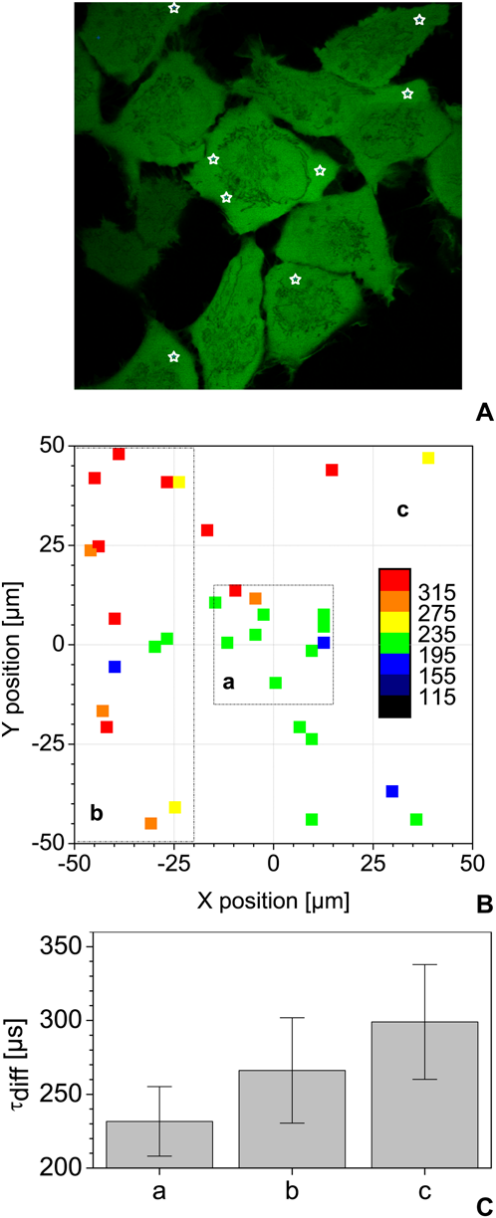
Effect of XY focus position on live cell measurements. Diffusion time of free eGFP was measured in the cytoplasm of living cells at various scanner X and Y positions as depicted by the white stars in (A). Several such acquisitions over a 100×100 µm area were performed and analyzed. The resulting diffusion times of 33 points in about 20 cells – after sorting out cells that had moved during the measurement – were color-coded as shown in (B) and subdivided in 3 distinct regions. Panel (C) presents the mean diffusion time and standard deviation obtained in the 3 regions shown in (B): a) for a central region of 30×30 µm b) for the whole scanning area of 100×100 µm and c) for a band of 30×100 µm on the left of the scanning area. All measurements were carried out at 37°C, with the same laser power, using a water immersion objective with a NA of 1.2.

FFM is very flexible in steering the laser beam in three dimensions. However, this versatility may result in optical path differences that cause noticeable changes in shape and size of the focal volume. We first tested the effect of changing z position. As seen in figure 3A for 2, 20 and 200 µm, the depth has no large effect on the autocorrelation functions. A slight increase in the number of molecules as a function of z depth may be observed between 200 µm and the cover slip, but can be neglected compared to cell-induced heterogeneity (figure 3B) and [31]. In this range, the diffusion time does not show significant variations (figure 3C). On the other hand, it is highly dependent on the x-y position of the focal point: this may lead to more than 100% systematic error and large variations in the results (figure 4A). Within a central area of 30 µm by 30 µm, however, reproducible measurements in solution are possible (figure 4B). To confirm this, we performed FCS measurements in the cytoplasm of live cells expressing eGFP (figure 5A, B). We then compared values obtained for the central region to the data obtained from points measured in the whole scanning field and to a band at the left of this area (figure 5, C) confirming that diffusion time is highly position-dependent when measured outside the central region.

### No fluorescent eGFP degradation products can be found in gels

**Figure 6:**
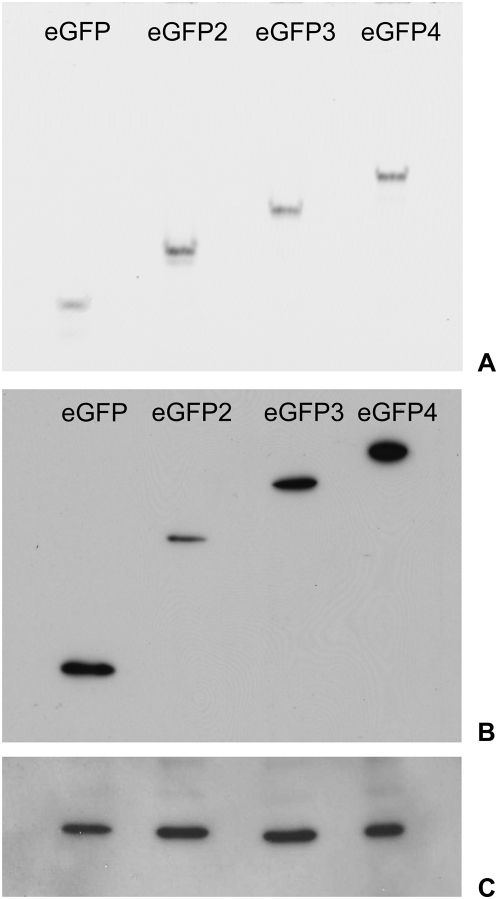
In-gel fluorescence of eGFP multimers. (A) Extracts of HeLa cells expressing the different eGFP multimers (mono- to tetramers, eGFP to eGFP_4_) were analyzed with non-reducing SDS-PAGE and a fluorescence reader. (B) Immunoblotting of the same extracts after reducing SDS-PAGE and use of an anti-GFP antibody. (C) Loading control with mouse monoclonal Anti-β-Actin antibody.

Fluorescence from the eGFP-constructs was found in the cytoplasm and in the nucleus of all observed cell lines. We characterized the *in vivo* expression and degradation products from the eGFP oligomers using native fluorescence gels and Western Blots, and analyzed the soluble protein extracts on native polyacrylamide gels. Figure 6A shows that the four eGFP constructs are separated according to their molecular size and that for every construct only the transfected fluorescent protein was found in the cells. Moreover, no fluorescent eGFP-degradation products show up on native gels, as found previously in [30] by an identical method.

Samples were further characterized by immunoblotting, where we used the same soluble protein extracts as for native gels. Figure 6B shows the eGFP constructs separated by their molecular size with no observable eGFP degradation products in the cell extracts. Since the cells were lysed mechanically rather than chemically, we did not observe the non-fluorescent degradation products found in [30] by immunoblotting.

### Mobility of eGFP multimers in the nucleus

The recorded confocal images of cell nuclei show that unmodified eGFP is distributed through the whole cell and can diffuse into and out of the nucleus due to its low molecular weight of 27 kDa [42]. Further examination of the images shows a high degree of penetration of the different eGFP-constructs into the chromatin network, as previously observed with other probes [43]–[45]. The different eGFP-constructs do not accumulate or aggregate in specific regions of the nucleus, they are rather homogeneously distributed with a slight reduction near the nuclear membrane. Moreover, the eGFP concentration did not correlate with chromatin density. However, a significant decrease, practically resulting in exclusion, was seen in the nucleolus, as previously observed by fluorescent confocal microscopy in [30], by laser scanning microscopy in [46] and for other proteins [47]. The amount of eGFP oligomers in the nucleus also decreased inversely to their size, in agreement with previous reports [30], [46], [48].

**Figure 7:**
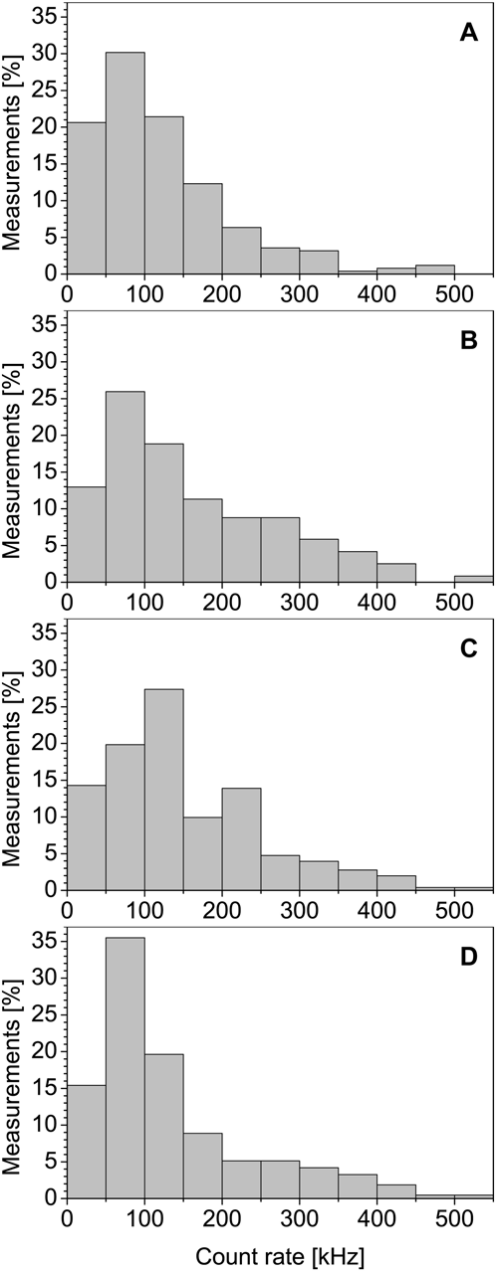
Count rate distribution of the eGFP-oligomers. The distribution of the measured count rates for the monomer (A), dimer (B), trimer (C) and tetramer (D) are nearly identical. Only the eGFP-tetramer shows an increased density of measurements in the range from 50 kHz to 100 kHz. These distributions imply that the number of molecules in focus decreases with increasing oligomer size, as the tetramer has a four-fold higher brightness than the monomer.

**Figure 8:**
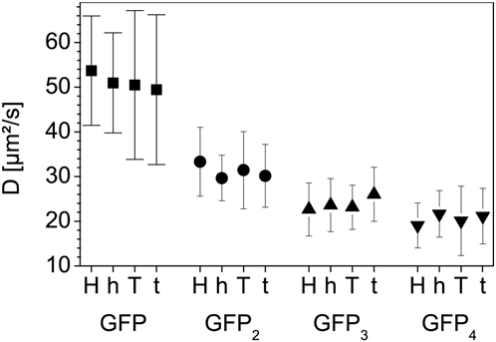
Diffusion coefficients of the eGFP-multimers. The figure shows the average diffusion coefficients with their associated standard deviation of the four eGFP oligomers in the nuclei of all four tested cell lines. H: HEK293, h: Hela, T: TP366, t: T98G cells.

Fluorescence fluctuation of the eGFP-constructs in the nuclei of interphase cells was recorded simultaneously with histone-mRFP1 fluorescence intensity. We measured the fluctuations of the eGFP-oligomer fluorescence at intensities ranging from 10 kHz up to 550 kHz for every constructs. Figure 7 shows the distribution of the intensity resulting from the eGFP_n_-fluorescence for all measurements. To avoid the ambiguity commonly associated with histogram representations (i.e. the bin size), we choose the bin size using the formula proposed by Scott and given by *W* = 3.49⋅σ⋅*N* ^−1/3^ [49], with *W* the width of the histogram bin, *σ* the standard deviation of the distribution and *N* the number of available samples. To allow comparison between the distributions we chose to hold the bin size *W* identical at the smallest calculated bin size for all 4 histograms. The distribution of the measured count rates is very similar for the four constructs, with only the tetramer showing a density increase in the range from 50 kHz to 100 kHz. As the distributions are quite similar for all constructs, it follows that the number of molecules in the focus decreased with increasing molecular size of the eGFP-oligomers, because the tetramer shows a four fold higher molecular brightness than the monomer. Resulting from this, one could expect increased variations in the diffusion coefficients due to a lower signal-to-noise-ratio. However, figure 8 shows that this effect is not present.

For each construct expressed in the 4 tested cell lines, we obtained the diffusion times and the relative amplitudes of two diffusive components. The diffusion times (ι_diff_) were normalized and converted to a diffusion coefficient using the calibration values of Alexa 488 on each measurement day. The diffusion coefficients of the eGFP-multimers were always in the same range for a given construct and no statistically significant differences could be observed for the distributions of one construct type between the different cell lines (fig. 8). Moreover, the different oligomers could well be differentiated from each others according to their diffusion coefficients.

Without differentiating between cell lines, the diffusion coefficients show a distribution between 87 and 20 µm^2^ s^−1^ (50.6 µm^2^ s^−1^ average) for the EGFP-monomer, between 58 and 14.5 µm^2^ s^−1^ (31 µm^2^ s^−1^ average) for the dimer, between 44 and 9 µm^2^ s^−1^ (23.8 µm^2^ s^−1^ average) for the trimer and between 42 and 8 µm^2^ s^−1^ (20.2 µm^2^ s^−1^ average) for the tetramer (data not shown).

**Figure 9:**
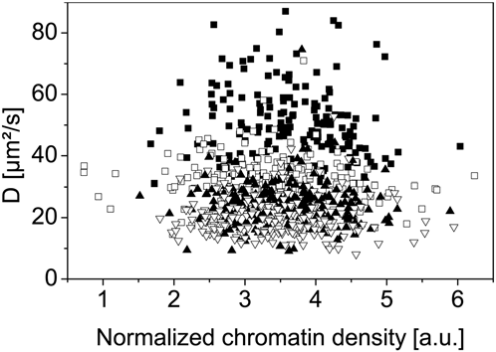
Overview of the diffusion coefficients of every eGFP construct in each cell line. The figure shows all the measured diffusion coefficients of the eGFP oligomers in the cell nuclei plotted against the normalized chromatin density, given by the H2A-mRFP1 fluorescence intensity. Black squares: eGFP-monomer, white squares: dimer, black triangles: trimer, white triangles: tetramer.

**Figure 10:**
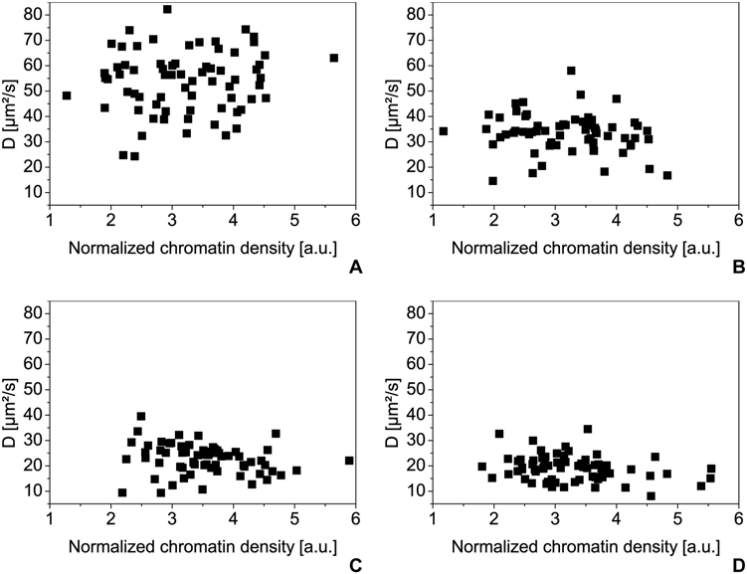
Diffusion coefficients of the eGFP-oligomers in HEK293 cells. The diffusion coefficients in HEK293 cells are plotted against the normalized chromatin density given by the H2A-mRFP1 fluorescence intensity for the eGFP-monomer (A) (70 samples), the dimer (B) (63 samples), the trimer (C) (66 samples) and the tetramer (D) (67 samples). All measurements were carried out at 37°C in 5% CO_2_ atmosphere, using a water immersion objective with a NA of 1.2.

For analyzing a possible correlation between the mobility of the proteins and the chromatin density, we plotted the normalized diffusion coefficients of the fast component against the normalized chromatin density for each cell line (fig. 9). First of all, we observe that we can distinguish the monomer, dimer and trimer populations from each other in every cell line; the diffusion coefficients of the trimer and the tetramer however do not differ significantly. In all cell lines the diffusion coefficient is independent on mRFP1 intensity and therefore on the chromatin density. Figure 10 present such results obtained from HEK-293 cell lines expressing eGFP monomers (fig. 10A), dimers (fig. 10B), trimers (fig. 10C) and tetramers (fig. 10D).

Similarly, the diffusion times of the slow component and the relative proportions of the fast and slow components show variations, but no dependence on the chromatin density when plotted against the normalized mRFP1-intensity (data not shown). Thus, we can assume that on the length scale of the resolution of the experiment (focus diameter about 0.3 µm) there is no correlation between the diffusional behavior of proteins in the range from 27 to 108 kDa and the average local chromatin density.

### Diffusional behavior of eGFP-oligomers

**Figure 11:**
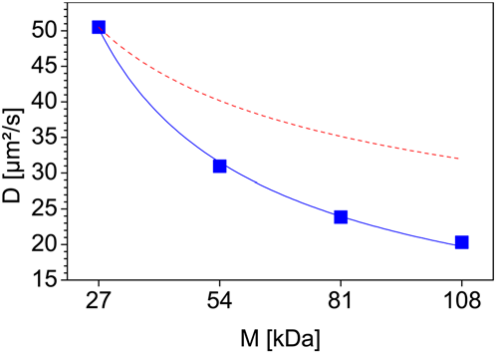
Decrease of the diffusion coefficient in the cell nuclei. The figure shows the diffusion coefficients of the eGFP oligomers (blue squares) plotted against their molecular weight. The diffusion coefficient shows a stronger decrease in cell nuclei (blue curve) than expected for free diffusion (red dashed curve). The power-law has here an exponent of −0.67 for the diffusion of the eGFP oligomers while free diffusion would show −0.33.

The diffusion coefficient of the eGFP-oligomers decreases from the monomer to the tetramer much more strongly than expected for free globular particles in solution (fig. 11). The radius of a globular particle is proportional to the cube root of the molecular weight (MW), thus its diffusion coefficient should be proportional to MW^−1/3^. However, figure 11 shows a power-law size dependence of the diffusion coefficients with an exponent of −0.67, while normal free diffusion in solution would show −0.33 (dashed curve on fig. 11). Thus, either the chromatin network or other nuclear structures are obstructing the diffusion of the eGFP-multimers, or the eGFP-multimers do not have a globular shape.

**Figure 12:**
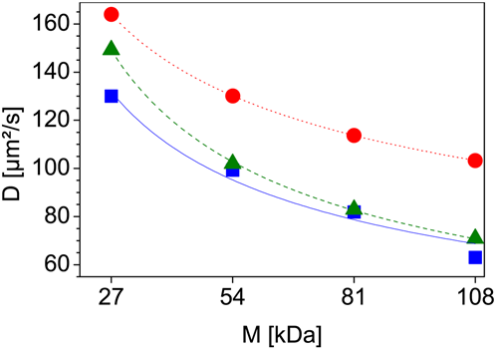
Free diffusion of the eGFP-oligomers in solution. The squares and the solid curve (blue) show the measured diffusion coefficient for the different eGFP-oligomers, while the circles and the dotted curve (red) represent the theoretical values for a globular shaped molecule and the triangles and dashed curve (green) for a rod-shaped molecule, plotted against the molecular weight of the different oligomers. All measurements were carried out at 37°C using a water immersion objective with a NA of 1.2.

To verify the molecular weight dependence of the eGFP-oligomers, we tested their diffusion coefficients free in solution. The measurements were carried out at 37°C so the diffusion coefficients could directly be compared to those in cell nuclei and temperature-dependent conformational changes could be excluded. The samples exhibited no unusual fluorescence bursts or other indicators of aggregation or contamination during the measurements. The autocorrelation curves of the different eGFP constructs were fitted to a one-component model. The resulting diffusion coefficients, together with the fitted diffusion curve (rectangles and solid curve), are shown in Fig. 12, as are the theoretical values for proteins with sizes of 27, 54, 81 and 108 kDa and globular shape (dots and dotted curve) or rod shape (triangles and dashed curve). The measured values are in good agreement with the computed diffusion coefficients for a rod shape. The diffusion coefficient of the eGFP-monomer is in good agreement with previously published values obtained by Scanning Fluorescence Correlation Spectroscopy [36] after correction for temperature of 37°C and by FCS in [50] after correction for the wrong diffusion coefficient of the reference standard. It also is in good agreement with the value obtained from raster image correlation spectroscopy [51]–[53] after correction for temperature of 37°C. Furthermore, diffusion coefficients of the different eGFP-homomultimers match those previously obtained by FCS published in [46] after correction for the wrong diffusion coefficient used there for the rhodamine 6G standard.

### eGFP diffusion maps

**Figure 13:**
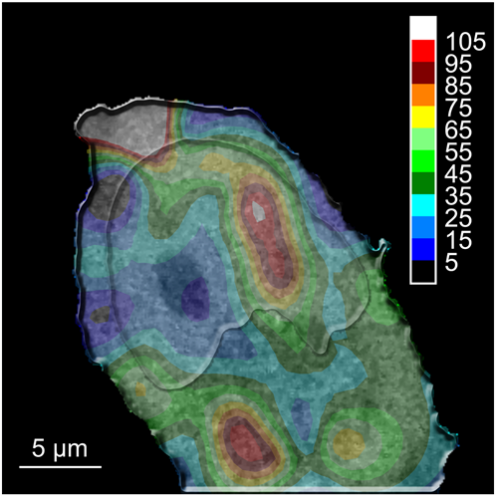
Interpolated diffusion map of eGFP in a HeLa cell. The map presents a combination of eGFP diffusion with the associated underlying confocal image of the H2A-mRFP fluorescence. The colors correspond to the diffusion coefficient of eGFP, and each color step equals a 12.125 µm^2^ s^−1^ range. Regions with different diffusion times can be observed in both the cytoplasm and the nucleus, as a visual lack of correlation between the chromatin density and the diffusion times. Measurements were carried out at 37°C in 5% CO_2_ atmosphere, using a water immersion objective with a NA of 1.2.

A rigorously characterized FFM system allows determining diffusion coefficients at any position of the living cell. It is thus possible to acquire a complete “diffusion map”. We present here such a map constructed from 50 localized measurements (2 times 3 s acquisition per measurements) performed on HeLa cells expressing free eGFP and mRFP1 tagged H2A-histones (figure 13). The map shows regions of homogeneous diffusion times both in the nucleus and in the cytoplasm. Different structures also emerge from the diffusion map in both the nucleus and the cytoplasm, as previously shown in [54] by time-correlated single-photon counting.

Figure 13 presents a diffusion map of eGFP diffusion combined with the corresponding H2A-mRFP confocal image. This further demonstrates the absence of visible correlation between chromatin density and eGFP diffusional behavior.

## Discussion

The fluorescence observed in the cells originates only from the complete eGFP-constructs, as previously stated in [30] and shown here with the help of native gels and immunoblotting. The observed penetration of eGFP-multimers into the cell nuclei and the fact that homomultimeric forms of eGFP can still diffuse into the nucleus, albeit with reduced import efficiency for increasing eGFP-multimer size, agrees with previous reports [30], [48]. We also could confirm the penetration of eGFP-tetramers into the nucleus as described by fluorescent confocal microscopy in [30] which, however, contradicts the results obtained by confocal laser scanning microscopy imaging in [48]. Our results, combined with the diffusion measurements of eGFP-multimers in solution, allowed us to draw conclusions about the structure of the eGFP-homomultimers. A rod-like structure of the multimers was proposed in [30] among other hypotheses to explain their ability to penetrate the cell nucleus despite of their size and the 50 kDa threshold of the nuclear pores. We were able to demonstrate here, by comparing free solution measurements with the theoretical dependence of simple structural models, that the diffusion properties of the eGFP-multimers are indeed best described by a rod-like shape, thus confirming the hypothesis stated in [30].

We showed that the mobility of eGFP and its oligomers (from about 27 to 108 kDa) in the interphase nuclei of living cells does not correlate with the chromatin density and that all of the intranuclear space, except the nucleoli, is freely accessible to such small proteins. This clearly indicates that even in its most compacted form, chromatin is not dense enough to significantly exclude proteins smaller than 100 kDa nor impede their diffusion, in good agreement with results obtained by single molecule tracking with high-speed fluorescence microscopy [47].

**Figure 14:**
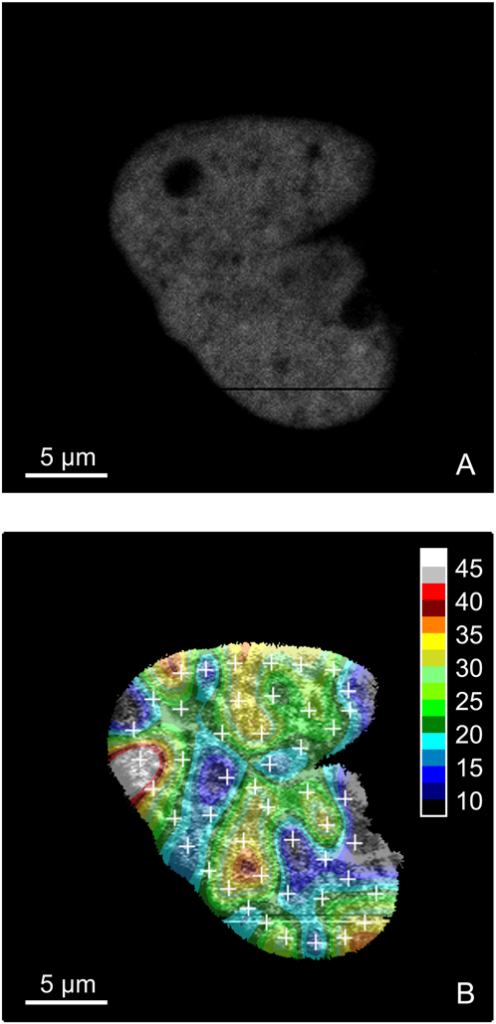
Diffusion map in the nucleus of a HeLa cell. Only the nucleus of a HeLa cell is represented on this figure and the color map shows the diffusion coefficient of eGFP in the nucleus. (A) Fluorescence intensity of H2A-mRFP1. (B) Diffusion map of eGFP (colors) underlaid by H2A-mRFP1 fluorescence intensity. The white crosses show the 48 measurement points, corresponding to a total measurement time of 288 seconds. Measurements were carried out at 37°C in 5% CO_2_ atmosphere, using a water immersion objective with a NA of 1.2.

**Figure 15:**
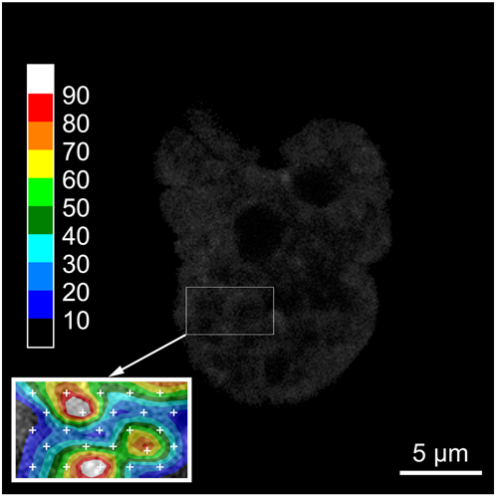
Interpolated detailed diffusion map of a part of a nucleus of a HeLa cell. The figure shows the H2A-mRFP1 tagged nucleus of a HeLa cell. Only the region in the cartridge is mapped and enlarged 2 times. Data were sampled on 25 points (white crosses in the cartridge), distributed on 5 lines of 5 points, with an inter-point distance of about 1 µm and 0.6 µm for the inter-line distance, covering an area of about 11 µm^2^. Each color step corresponds to a 10.0 µm^2^ s^−1^ range of the diffusion coefficient of eGFP. The white crosses mark the measurement points. Measurements were carried out at 37°C in 5% CO_2_ atmosphere, using a water immersion objective with a NA of 1.2.

This work also demonstrates that the dependence of the diffusion coefficient on molecular weight is greater, both in the cell nucleus and in solution, than expected from a free diffusion model for globular proteins. This implies obstruction of the free diffusional movement, either by a network with mesh size of the order of the size of the diffusing proteins, or by transient binding to nuclear structures. At any rate, nuclear structures different from the chromatin network must also affect the mobility of the eGFP probes since we do not find a correlation between the diffusional behavior and chromatin density. If a dependence on chromatin structure exists, it manifests itself probably on a length scale smaller than the resolution of the FFM technique (about 0.3 µm laterally and 1.5 µm vertically). Figure 14 further confirms this observation: one notices that there is no visible correlation between the diffusion of eGFP (Fig. 14B) and the local chromatin density (Fig. 14A). More detailed diffusion mapping, as seen in figure 15 for a part of a nucleus of a HeLa cell, higher resolution microscopic techniques such as STED and detailed studies of diffusion near nuclear structures like the lamina at the nuclear envelope or in the nucleoplasma (also known as the nucleoplasmic veil [55]) should help answering the remaining question of obstructed diffusion in living cell nuclei.
